# An overview of nutritional profiling in foods: Bioanalytical techniques and useful protocols

**DOI:** 10.3389/fnut.2023.1124409

**Published:** 2023-03-21

**Authors:** Deb Duhita Mondal, Ushashi Chakraborty, Manotosh Bera, Subhrojyoti Ghosh, Debasish Kar

**Affiliations:** ^1^Department of Biotechnology, Heritage Institute of Technology, Kolkata, India; ^2^Department of Biotechnology, Ramaiah University of Applied Sciences, Bengaluru, India

**Keywords:** nutrition, nutritional profiling, chromatography, microscopic, health, consumers

## Abstract

Maintaining a nutritious diet is essential for humans if they want to live a healthier life. Several food businesses and food safety organizations play a significant role and offer useful ways for improving nutritional quality that assists consumers in making informed selections. Making poor food choices and consuming unhealthy meals are the main causes of non-communicable diseases (NCDs). Nutritional profiling (NP) models are developed to evaluate the nutritional value, calorie content, and the amount of micronutrients and macronutrients contained in a given food accompanied by additional details on the nutritional anomaly provided by published standard nutrients and nutritional databases. To construct an ideal nutritional model that can facilitate food consumption, bioanalytical methods such as chromatography, microscopic techniques, molecular assays, and metabolomics can be applied. With the use of these technologies, one can learn more about the health advantages of nutrition and how to prevent disease. A wider element of NP is also provided by the developing technologies in the area of nutrition research, such as nanotechnology, proteomics, and microarray technology. In this review, we are focusing on the different bioanalytical techniques and the various protocols of NP and their application and refinement of the models. We have evaluated various NP techniques currently used in the food industry for the detection of different components present in food items.

## 1. Introduction

**Figure 1:**
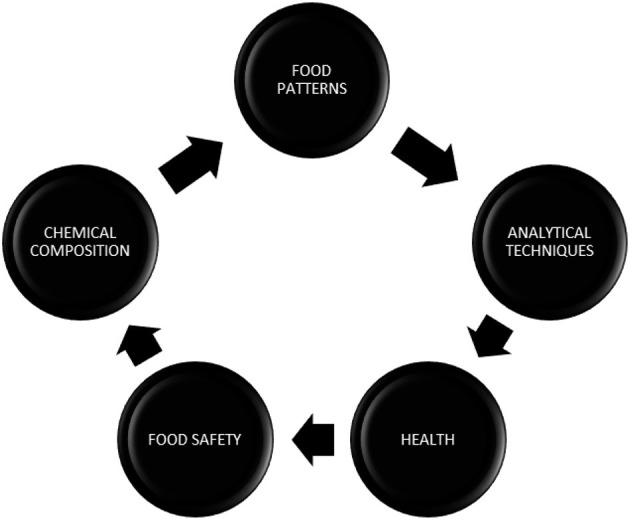
Nutritional profiling. The interrelationship of all the components that involve the development of the nutritional profiling model shows their interdependence with each other.

The primary cause of any non-communicable diseases (NCDs) across the world, with most cases in European countries, is an unhealthy diet, among other risk factors. An unbalanced diet could be established by taking into account many factors. In the last few decades, there has been a noticeable rise in overeating, and by 2025, it is predicted that one-fifth of persons worldwide will be obese. Finding and obtaining healthy foods will be challenging due to a variety of circumstances, including finances, individual preferences, different cultural traditions, geographical regions, and other environmental concerns such as climate change. The vast population lacks a proper dietary plan and instead consumes processed foods that are rich in fats, carbohydrates, and sodium (1). According to the WHO European Food and Nutrition Action Plan, food consumption that is high in energy or low in micronutrients and non-alcoholic drinks ought to be limited to a balanced diet to meet food targets for the vast population. At a conference held in 2020, health promotion and prevention of NCDs were promised, along with the development of common policies and implementation of standardized nutrition profile models (Figure 1). The nutritional value of a given product has been evaluated using a variety of nutritional profiling (NP) models based on its nutrient composition.

Several food firms have formed their nutritional standards by developing various models to inform the development of their products or highlight their healthy options (2). The nutrient profiling models, created for evaluating the nutritional content of meals, have developed into a crucial instrument for public policy. The energy density and the nutritional density of food are typically inversely correlated. NP models seek to discover nutritious foods that are high in nutrients and classify them based on their nutritional value. In developed nations, the nutrition profile models provide the scientific groundwork for a variety of educational, labeling, regulatory, and tax policies. Numerous front-of-pack icons and logos that convey a specific message are based on NP models (3).

The Food and Agriculture Organization of the UN (2007) strongly approves the consumption of certain food groups that have been associated with health-protective benefits, including fruits and vegetables, whole grains, legumes, and nuts. In contrast, studies on nutrition over a decade confirm the increased intake of beneficial nutrients such as vitamins, minerals, and fibers. The reason for this is the nutrient profile and the most common labeling schemes that provide nutritional information on the front of food packages, reducing the consumption of such unfavorable foods (4). EFSA's Panel on Nutrition Products and Nutrition and Allergies found that the NP of certain foods did not comply with reference intakes of nutrients. The comparison of the nutritional value of all foods and communicating their importance is made possible by the declaration of energy, macronutrient and micronutrient content that is established, and its relevant reference values among the general population (5). Finally, the strategy of using nutrient profiles is detrimental to some specific foods for which it is impractical to reconstitute in order to meet the threshold set for the same profile, resulting in being labeled “unhealthy”, and despite being present in significant amounts, it plays a beneficial nutritional role.

## 2. Need for nutritional profiling

Several definitions have been developed over the years due to the potential of NP as a tool for evaluating specific foods in terms of their contribution to healthy eating patterns. The generally accepted definition, put forth by WHO in 2015, is “the science of classifying or ranking foods following their nutritional makeup for reasons linked with disease prevention and health promotion.” NP is a phrase that has been connected to the composition of a food or diet in a context that is a helpful tool to help customers select healthier foods for themselves (6). The NP model can be used to improve the nutritional quality of diets by identifying foods that may constitute healthy and unhealthy diets and assessing the nutritional quality of foods rather than diets. Nutrient profiling is now used in a variety of nutrition policy applications worldwide, and the diversity of NP models has increased significantly in recent years. NP models are being used for a variety of purposes, such as helping customers choose healthier foods through food labeling systems, deciding which food items should be sold in schools, developing regulations for health or nutrition claims, and limiting the marketing of food to children (Table 1). The proliferation of NP models around the world, together with their numerous applications and specificities, might raise the likelihood of model differences and confuse regulators, producers, and consumers. The main goals of this article are to describe the process of developing an NP model and the various methods used for its validation, as well as to identify the potential role of nutrient profiling applications in promoting healthier food choices. This is done while also taking into account initiatives established in recent years concerning the development and application of various nutrient profiling tools. This cutting-edge review aims to improve the knowledge of NP models and assist policymakers in selecting an appropriate model once the implementation of nutrition-related policies would benefit from the use of such models. NP models serve as an excellent tool for decision-making in the area of public health nutrition interventions in order to help consumers choose healthier foods.

**Table 1:** Several schemes developed for nutritional model implementation across the world.

**Schemes**	**Food categories**	**Aim**
Pan American Health Organization model (USA)	Mostly processed and ultra-processed foods	Classifies foods and beverages based on excessive sugar content and fats and serves various strategies
Health Star Rating (Australia and New Zealand government)	Retail foods and beverages	Allow food comparison
Multiple traffic Light (United Kingdom)	Processed foods	Provides clarity on food characteristics
Mexican Committee of Nutrition Experts (Mexico)	Basic and non-basic foods	Selection of healthier food options

## 3. Analytical techniques for nutritional profiling

### 3.1. Chromatographic techniques

The discrimination of proteins with extreme physicochemical features is hampered by poor throughput, very restricted dynamic range, and other severe restrictions of conventional analytical methods. As a result, methods based on chromatography have been developed for the pre-separation of proteins and peptides. Chromatography is a laboratory technique for the separation of a mixture into its components. The combination passes through a system (a column, a capillary tube, a plate, or a sheet) in which a substance known as the stationary phase is fixed after being dissolved in a fluid solvent (gas or liquid) termed the mobile phase. The name of the method is either due to gas–solid chromatography or gas–liquid chromatography depending on the nature of the stationary phase used. The components of the mixture travel at varying apparent velocities in the mobile fluid, which causes them to separate because they tend to have different affinities for the stationary phase and are held for various amounts of time based on their interactions with its surface sites. The differential partitioning between the mobile and stationary phases serves as the foundation for the separation. This partition coefficient can be expressed as K_x_ = [C]_s_/[C]_m_, where [C]_s_ and [C]_m_ are concentrations of stationary and mobile phases, respectively. Chromatography can be used at different points in the food chain to assess food quality and find additives, pesticides, and other dangerous contaminants. Chromatography enables the food business to deliver precise details about the nutrients in a specific product as well as many other things (7). Many food businesses use various additives and preservatives to try to improve their manufacturing process, which necessitates additional testing procedures to guarantee the safety of their goods. However, chromatography is by far the most adaptable technique, and many businesses can perform the majority of the necessary tests using chromatography equipment.

#### 3.1.1. Gas chromatography

**Figure 2:**
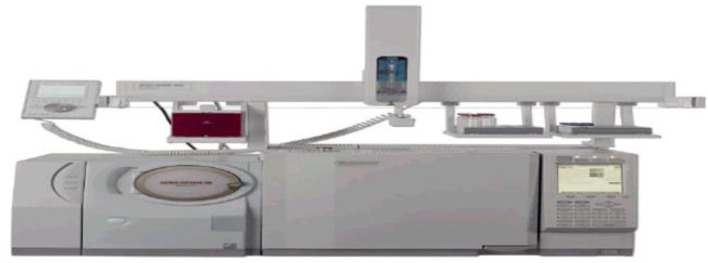
Gas chromatography. A typical GC-MS tool is used for the analysis of food for determining the food quality and evaluating food's nutritional benefits.

The earliest established chromatographic technique, gas chromatography (GC) analysis, which is being used today, forms the foundation of a traditional method for evaluating the quality of food. GC is a popular method of chromatography used in analytical chemistry for separating and studying substances that may be evaporated without decomposing. It is frequently used to determine a substance's purity or to separate the various ingredients in a combination. GC may be used in preparative chromatography to separate pure substances from a mixture. An inert or nonreactive gas continuously flows down a small tube known as the column, which is the foundation of a gas chromatograph, carrying the vaporized sample through it. Depending on their chemical and physical characteristics and the interactions they have with the stationary phase, the filling or lining of the column, various components of the sample move through it at different speeds. The popularity of gas chromatography is due to the unique combination of its high sensitivity, wide dynamic concentration range, excellent selectivity, and resolution (8). GC is widely utilized to research compounds such as sterols, oils, low-chain fatty acids, aroma components, and other contaminants such as different pesticides, pollutants from the industry, and particular classes of medications in food (Figure 2). Food quality is determined by its origin, chemical composition, adequate physical properties (e.g., texture, color, and tenderness), unique sensory evaluation, and several precautionary norms regarding toxic and microbiological contamination (9). In GC, the components present in the sample to be injected are constantly pushed through the column by a mobile gas phase, allowing them for separation and eluting through the column outlet. The retention times vary with phase loading, temperature, and flow; each probe's retention index is calculated by infusing the probe compound with a group of regular hydrocarbons that will span the compound's retention time. The retention index can be calculated from the following equation:


(1) $$\begin{array}{l} I=100z+100\frac{\log\left(t_{R\left(x\right)}^{1}-t_{R\left(z\right)}^{1}\right)}{\log\left(t_{R\left(z+1\right)}^{}+t_{R\left(z\right)}^{1}\right)} \end{array}$$

In the abovementioned equation, t'_R_ is the adjusted retention time and both x and z are the number of carbon that are present in the hydrocarbon before the sample elution; (z+1) denotes the hydrocarbon after elution. Both the gas and the sample then pass *via* a detector through the column. The apparatus produces an electrical signal and measures the sample's volume. A chromatogram is produced by a signal of the detector and is used for gathering and analyzing samples for qualitative and quantitative data (10). Food samples must be homogenized as part of sample preparation to effectively extract the metabolites and minimize experimental mistakes. To extract the non-volatile, hydrophilic, low-molecular-weight molecules, such as amino acids, sugars, and organic acids, an extraction solvent is next added to the sample. Methanol is frequently used in methods to extract polar molecules. In some research, liquid extraction with methanol and chloroform is frequently used (11). Water impedes the derivatization reaction; therefore, after extraction, the samples are freeze-dried to eliminate it. The separated compounds are derivatized to increase the polar molecules' volatility for GC. The derivatization procedure uses oximation based on methylamine, silylation based on N, O-bis(trimethylsilyl)trifluoroacetamide, N-methyl-N-trimethylsilylacetamide (MSTA), and/or trimethylchlorosilane. After being derivatized, the sample is placed into a GC vial for GC/MS analysis. Multiple peaks are present in the GC/MS raw data, which must be deconvoluted and recognized. Deconvolution distinguishes between the true metabolite peaks and noise signals, and the numerous peaks indicate the strengths of distinct metabolites. The annotation of identified peaks is then performed by comparing them with known spectra from a metabolite spectral library (12). Following data processing, both univariate and multivariate analyses will be possible using a data matrix comprising the identification and intensity of the metabolite. These studies can currently be carried out by many universal applications, including the MSDIAL software and the XCMS online platform.

#### 3.1.2. High-performance liquid chromatography

This method was initially an abbreviation for high-pressure liquid chromatography since early columns produced high operating pressures. High-performance liquid chromatography (HPLC), stressing the successful separations attained, had taken over as the favored phrase by the late 1970s. A discrete small amount (usually a few microliters) of the sample combination to be separated and studied is added to the stream of mobile phase percolating through the column. It has been widely recognized that polyphenolic compounds are a key dietary component for antioxidants that occur naturally and provide numerous health benefits. From a nutritional perspective, the need for quick, precise and sensitive techniques for food sample detection is in demand. For instance, creating food composition databases is the primary prerequisite for determining the daily intake of polyphenolic compounds. Phenolic compounds such as flavonoids, phenolic acids, tannins, stilbenes, and lignans are extraordinarily complex chemicals with a variety of health benefits and draw attention when they are examined in diverse dietary samples. The most popular separation technique for these uses is HPLC. Plant-based analysis of sugars with conventional extraction processes can create challenges due to the excessive absorption of water by these samples for subsequent separation by HPLC.

The sample's components flow through the column at various speeds as a result of their various physical interactions with the adsorbent (also called the stationary phase). Each component's velocity is influenced by its chemical makeup, the characteristics of the stationary phase (column), and the makeup of the mobile phase. The retention time of a particular analyte is the time at which it elutes (emerges from the column). The strength of elution is measured by the polarity index denoted by using P. The more the value of P, the higher the eluent strength. For example, the polarity index of a solvent P_m_ composed of solvents a and b, respectively, is P_m_ = ${\text{P}}_{\text{a}}^{*}$ X_a_ +P_b_^*^ X_b_, where P_a_ and P_b_ are polarity indexes of solvents a and b, respectively, and X_a_ and X_b_ are the fractions of their volumes. The effect of eluent polarity on the capacity factor k' of a compound is given by the equation:


(2) $$\begin{array}{l} \frac{{{\text{K}}^{\text{′}}}_{2}}{{k^{\prime }}_{1}}=\frac{10\left({P^{\prime }}_{2}-{P^{\prime }}_{1}\right)}{2} \end{array}$$

(3) $$\begin{array}{l} \frac{{{\text{K}}^{\text{′}}}_{2}}{{k^{\prime }}_{1}}=\frac{10\left({P^{\prime }}_{1}-{P^{\prime }}_{2}\right)}{2} \end{array}$$

where P_1_' and P_2_' are the polarity indices of the two eluent mixtures. HPLC has a few advantages over traditional low-pressure column liquid chromatography methods such as speed, as many studies suggest that an operation can be completed within 30 min; diversification, as different detectors can be employed to improve resolution and sensitivity; and lastly recovery of a sample due to the low volume of eluent (13). Another application of HPLC, which is frequently employed for the separation and purification of macromolecules like proteins and polysaccharides, is the evaluation of tiny molecules and ions such as sugars, vitamins, and amino acids (14).

The characterization of major and minor sugars relevant to nutrition can now be accomplished using HPLC. A minute change in the mobile phase flow rate can characterize the presence of sugar concentration in food (15). In addition, HPLC is utilized in distinct domains of carbohydrate research, including enzyme investigations on polysaccharides and their further analysis providing food makers with a quick quantification method with good accuracy and reproducibility. Screening for the presence of sucrose, maltose, glucose, and fructose in high-protein ingredients derived from legumes, pseudocereals, and cereals, such as quinoa, amaranth, and buckwheat as well as from soy, pea, lupin, lentil, carob, chickpea, and fava beans, confirmed the suitability of the selected extraction technique (16).

There is a significant need for accurate information about the amount of vitamin K present in food and feed products, as well as in human and animal blood, to better understand the vitamin's nutritional role. We concentrate on the vitamin phylloquinone, often known as vitamin K1, which is produced in plants due to its significance. The function of the so-called menaquinones, which are present in microbes, is currently unknown. These days, HPLC defeats time-consuming bioassays and thin-layer chromatography as the best analytical approach. Electrochemical or fluorescence detection provides the necessary results for the food components followed by the cleaning of samples. In the case of fluorescence detectors, the equation for dilute solutions can be written as I_f_ = I_o_φ_f_ (2.3abC), where I_f_ is measured emission intensity; I_o_ is excited beam intensity; φ_f_ is the number of photons emitted; a is the molar absorption coefficient; b is the cell path length; and C is the concentration of the sample. A normal-phase HPLC is sometimes employed as a solid-phase extraction step. A straightforward, quick, and adaptable HPLC assay was introduced to replace time-consuming and labor-intensive methods for determining vitamin K1 (phylloquinone) (17). It should be stressed that affordable chemicals and generally accessible laboratory equipment were used. Materials of various origins were examined, and it was determined that the process was suitable for these ends. In the future, further research on NP will provide insight into a better-determining factor for the composition of foods and nutritional databases.

#### 3.1.3. Chromatography in determining vitamins in food

Chromatographic methods developed over the past few decades have the capability of determining the vitamins having diverse chemical properties that are present in food, which are responsible for different biological activities in humans. The unstable nature of the target analytes makes routine analysis of vitamins problematic. Numerous elements, including exposure to heat, light, and air, as well as interactions with other dietary ingredients, can impact the stability of vitamins. Two qualitative techniques for the detection of water-soluble and fat-soluble vitamins were quickly developed combining reverse-phase high-pressure liquid chromatography with diode array detection (DAD). Due to the instability of different vitamins, whose breakdown frequently takes place during sample preparation, distinct HPLC procedures are advised for quantitative analysis.

Vitamins A, E, and D generally considered fat-soluble vitamins can be detected using HPLC-UV present in butter and vegetable oils. Vitamin C content, high in citrus fruit, is estimated using the reverse-phase HPLC method with the presence of UV at a certain wavelength, and optimized pH can also simultaneously determine citric acid, malic acid, and quinic acid present in fruits. Through the analytical procedure, vitamin B6 compounds can be determined in intact forms followed by extractions and assays, thereby performing a routine analysis in meat, fish, and other dairy food products (18).

Foods are subjected to vitamin analyses for a variety of reasons, such as regulatory compliance, nutrient labeling, and determining how food processing, packaging, and storage affect variations in vitamin content. HPLC is becoming a more significant method because of its automation capabilities employing autosamplers and robotics. Since the vast majority of vitamins are found in foods in tiny amounts, detection and sensitivity are important factors to take into account.

Fluorescence and electrochemical detection are also utilized in some circumstances, even though UV absorbance is the most typical detection technique. Due to its intrinsic lack of specificity and sensitivity, refractive index detection is not frequently utilized for vitamin identification.

Smaller particles, shorter columns, and microbore columns are being employed more frequently to increase speed and sensitivity in HPLC procedures that typically involve bonded phases, notably RP packing materials (19). Although UV and Fluoresence Detection (FLD) are frequently used, Electrochemical Detection (ED) is becoming more significant since it has improved sensitivity and selectivity for the detection of extremely minute levels of vitamins. The sample preparation process is made easier, and some oxidizable vitamins are shielded from oxidation by the use of HPLC column-switching procedures. For routine work in food control laboratories, where quick analysis and straightforward sample preparation are required, as well as a trustworthy and repeatable chromatographic test, these automatic techniques are becoming more crucial (20).

#### 3.1.4. Chromatographic techniques are useful for identifying adulterations

Food adulteration is the deliberate lowering of a food's quality by the addition or substitution of unapproved alternative ingredients, the removal of important ingredients, or both. This is typically done to reduce the price or boost the volume of a certain food product. Chromatography-based techniques may identify various food adulterations. Among the most popular analytical detection techniques were HPLC and GC. With the use of these procedures, which are able to undertake qualitative and quantitative analyses of many classes of food elements, almost all foods may be evaluated.

### 3.2. Spectroscopic techniques

The dispersion of light into its individual colors is referred to as spectroscopy. It is a technique used for determining how much light is absorbed by a chemical material and at what intensity light flows through it. Spectroscopy is regarded as a crucial technique employed in the NP of food, both qualitatively as well as quantitatively. Spectroscopic methods help determine protein interactions and can be used with or without the combination of other analytical methods. The key principle is supported by electromagnetic light radiation being absorbed, transmitted, and emitted and the nature of its interaction with molecules, following the theory of effective collision. Since light itself is electromagnetic radiation, it can be useful for detecting the presence of microbes, harmful pathogens, and other compounds present in food and their analysis thereafter. Hence, food quality is ascertained through spectroscopic techniques, which can quickly distinguish the carbohydrate, fats, and water available in a variety of foods.

#### 3.2.1. NMR spectroscopy

**Figure 3:**
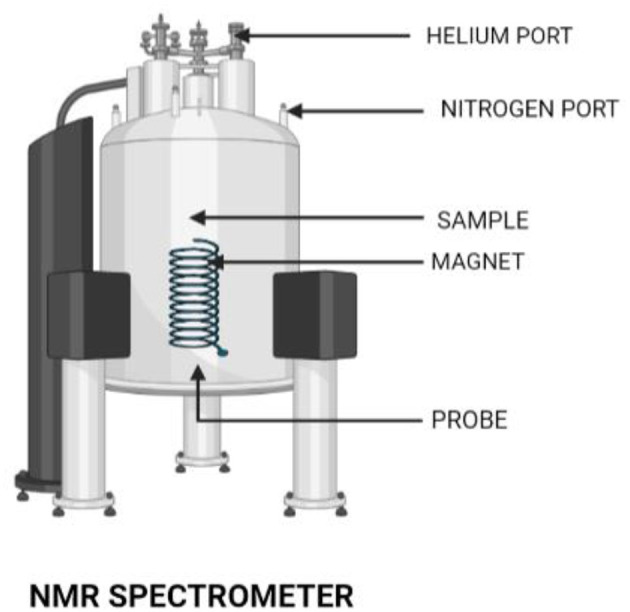
NMR spectrometer. An NMR spectrometer has a wide application in the field of research. This tool is also used in identifying genetic traits of foods.

An important spectroscopic method for determining and calculating water content in food items is nuclear magnetic resonance (NMR), an experimental procedure that calls for meticulous calibration. The interaction between the magnetic field used and the magnetic characteristics of atoms and molecules is utilized by NMR spectroscopy (Figure 3). The fact that the resonance frequency of a given sample substance is precisely proportional to the intensity of the applied magnetic field is a crucial aspect of NMR. The resonance frequencies of a sample's nuclei depend on where in the field they are situated if it is placed in a non-uniform magnetic field, and imaging techniques make use of this fact. Three successive processes typically make up the NMR principle:

- The polarization of magnetic nuclear spins in a static magnetic field (B_0_) that is being applied.
- The nuclear spin alignment is disturbed by a radio frequency (RF) pulse, which is a weakly oscillating magnetic field. The static magnetic field (B_0_) and the observational nuclei affect the oscillation frequency necessary for meaningful disruption.
- The precession of the nuclear spins around B_0_ causes a voltage to be created in a detecting coil, which in turn causes the NMR signal to be detected during or after the RF pulse. Precession after an RF pulse often takes place at the nuclei's intrinsic Larmor frequency and does not, in and of itself, require changes in energy or spin states.

Food ingredients can be measured using this specific type of spectroscopic technique for a variety of applications. NMR is capable of identifying the genotypic features of grapes that are preserved to grow wine. In addition, it can also examine when food products have been adulterated, ripened, or dried out. In instances where sample composition is unknown, NMR can reveal the metabolic components of the sample. To identify biotic and abiotic stress in plants as well as genetic variations, the NMR spectroscopy technique is performed in conjunction with a multivariate analysis. This particular method is frequently used to detect metabolites present in fruit juices, wine, tomatoes, and beer as well as flavonoids, organic acids, and soluble sugars. An added advantage here is the fact that NMR equipment used in NP is portable. Due to the NMR spectra generating a significant quantity of data, its results are evaluated through Student's *t*-test and variate analysis. However, the identification of a molecule is at times proven to be difficult owing to the overlapping of spectra.

#### 3.2.2. Atomic absorption spectroscopy

Atomic absorption spectroscopy (AAS) is an instrumental analytical technique used for quantifying trace elemental concentrations in a sample. This method employs light absorption of free atoms that are in gaseous form, and the measurement of the concentration of a particular analyte is done based on how much specific light has been absorbed in a certain wavelength. It is one of the foremost valuable means to identify nutritional constituents in food items such as iron, sodium, and calcium. AAS is essential as the analysis of trace elements forms an integral part of the labeling and quality control of food items.

The most recent AAS makes use of fiber optic technology, which results in an entirely enclosed optical system. Better light throughput for higher detection thresholds is provided by the optical system. The instrument's size is similarly decreased by the improved light path. In addition, it employs a layered architecture that enables the employment of a graphite furnace and flame on a single instrument. It makes use of a burner made of titanium which is simple to remove for various analyses. For quick startup and stability over time without the requirement for recalibration, it has a twin-beam design. Analyzing food products by AAS has novel applications, like the identification of infant food samples and formula, vegetables and oils derived from them, and meat and filets of fish (21).

#### 3.2.3. Mass spectrometry

It is an experimental method for calculating the atomic or molecular weight of materials. At present, modern mass spectroscopic detectors used in mass spectrometry (MS) are the greatest ways to identify a wide variety of compounds. All molecules have a mass. As MS distinguishes chemicals present as ions in the gaseous phase, it is difficult to achieve an accurate measurement of a chemical spectrum. For ionization in the gas phase, the requisites vary greatly among chemicals. The energy needed to produce ions can cause chemical species to change as a result of interactions involving metabolites. Furthermore, because of the presence of other molecules, a single chemical can produce a variety of ionic forms with varying relative abundances. Standardized separation methods, such as liquid and gas chromatography and capillary electrophoresis, which reduce the complexity of the chemical mixtures entering the mass spectrometer, and techniques for ionization, such as atmospheric pressure chemical ionization (APCI), electrospray ionization (ESI), and desorption electrospray ionization (DESI), all of which ionize various chemicals, control these limitations. For the purpose of trace element detection and chemical speciation, HPLC is frequently used in combination with inductively coupled plasma-mass spectrometry (ICP-MS) owing to its efficient separation technique. HPLC-ICP-MS coupling is currently an indispensable screening method employed for the identification of unknown metal species as it quantitatively reacts to every molecule where a certain heteroelement is present, notwithstanding its coordination environment.

Absolute quantification by MS necessitates normalization concerning real standards and is frequently easily accomplished only for a few compounds. Nutritional metabolomics continue to face significant challenges due to the limited capacity to obtain the absolute calculation of a high number (>2,000) of metabolites. Based on mass resolution and accuracy, the high-resolution MS method has been utilized for estimating a huge number of compounds. Using precise mass/charge (m/z) values, these properties enable the elemental composition of a chemical to be predicted (22).

### 3.3. Polymerase chain reaction

**Figure 4:**
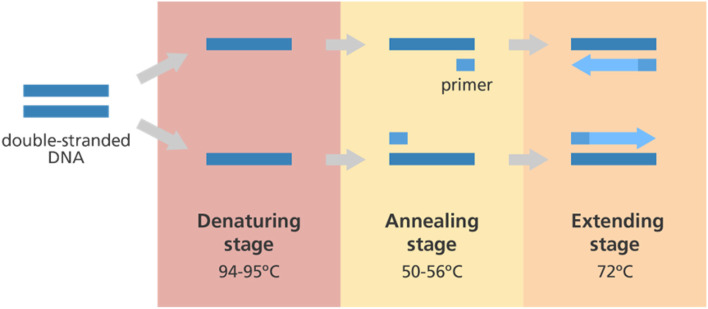
Polymerase chain reaction (PCR). A typical schematic representing the workflow for the PCR reaction. The double-stranded DNA undergoes three major steps, namely denaturation, annealing, and extension before the amplification step.

A handful or a singular nucleic acid sample can be amplified using the polymerase chain reaction (PCR) technique to produce thousands or even millions of copies of that same nucleic acid (Figure 4). This makes characterizing and comparing genetic material from various people and species simpler. Altogether, it is regarded as a machine for duplicating DNA on a molecular level. The fundamentals of the PCR technique are founded on heat cycling, which makes use of thermodynamics in interactions between nucleic acids. Thermal cycling of the PCR samples after a specified set of temperature increments is now used by most PCR machines. These thermal cycling stages are initially necessary for the physical separation of two strands in a dsDNA double helix before the high-temperature process of DNA replication. The DNA polymerase enzyme then helps in synthesizing the new DNA strands that are complementary to each other by using each of them as a template for dsDNA synthesis at lower temperatures. Traditional methods for detecting infections and other microbes rely on culturing techniques, but these are laborious and time-intensive, no longer meeting the expectations of diagnostic laboratories and quality control processes to deliver outcomes quickly. Direct identification of microbes in a food item is greatly expanded by the specificity and sensitivity of PCR, which can deliver precise results in roughly 24 h.

Some pathogenic DNA or RNA are mostly the main targets in the food products for evaluating food authenticity such as microbes causing spoilage, molds producing mycotoxins, toxin-generating bacterial DNA, and DNA containing unwanted trace components. Several issues can arise when PCR is used for the identification of pathogens in food products such as toxicogenic fungi, although many of them may be resolved by using appropriate sample preparation techniques. To detect dietary allergens like peanuts, buckwheat, and wheat, precise and sensitive real-time PCR approaches have been developed. These methods can balance instrument effects and reduce the possibility of false-positive and false-negative results (23).

Real-time PCR has emerged as a viable alternative method for food diagnostics. It has a variety of benefits over traditional culturing methods, including speed, exceptional analytical sensitivity and selectivity, and the ability to quantify. However, the actual application of it for food monitoring and control is being hampered by the use of expensive tools and reagents, the requirement for knowledgeable personnel, and the absence of established protocols.

For the identification of dietary allergens like wheat, buckwheat, and peanuts, precise real-time qualitative PCR approaches have been developed. These methods can balance instrument effects and reduce the possibility of false-positive and false-negative results. Using reference plasmids that contained known copies of the target sequences, the cutoff for identifying positive samples was established in each run of the real-time PCR assay. The allergenic components in highly processed foods (cooked for longer than 30 min at 122°C) corresponding to 10 ppm (w/w) protein were identified using the copy counts of the plasmids. A reference plasmid analysis for each real time PCR is run in reduced instrument and variability. In addition, it assisted in preventing false positives brought on by minute quantities of agricultural- or laboratory-related pollutants. Using 79 frequently consumed food items and some of their relatives, the specificity of the real-time PCR approach was confirmed. In various types of samples, the approach was found to be perceptive enough to identify allergenic components matching 10 ppm (w/w).

### 3.4. ELISA

The test employs a solid-phase form of enzyme immunoassay (EIA), utilizing antibodies against the target protein to find the presence of a ligand (often a protein) in a liquid sample. Antigens from the sample to be examined are coupled to a surface in the simplest ELISA. The surface is then covered with a corresponding antibody so it can bind the antigen. Any unbound antibodies are then taken out when this antibody is coupled to an enzyme. A substance containing the enzyme's substrate is introduced in the last stage. If there was binding, the next reaction generates a discernible signal, most frequently, a change in color. An ELISA requires at least one antibody that is specific for a given antigen. The sample containing an unknown quantity of antigen is either non-specifically (through adsorption to the surface) or specifically immobilized on a solid substrate (often a polystyrene microtiter plate) (*via* capture by another antibody specific to the same antigen, in a “sandwich” ELISA). The detecting antibody is added and forms a complex with the antigen once the antigen has been immobilized. The detection antibody may be bioconjugated to an enzyme or may be covalently attached to an enzyme in order to be detected by a secondary antibody. To get rid of any non-specifically bound proteins or antibodies, the plate is routinely cleaned with a mild detergent solution between each step. After the final wash process, an enzymatic substrate is added to create the plate, which provides a visual signal that indicates the quantity of antigen in the sample.

The serological methods like precipitation and agglutination frequently used in food microbiology to isolate fungal strains from bacterial ones are gradually being replaced by contemporary, precision immunoassays based on a format similar to ELISA. Although occasionally poisons, enzymes, or polysaccharides present extracellularly are detected, the majority of immunoassays are predicted by certain antigens being present in cell membranes or the cytoplasm. Several microbial species and strains for which there are commercially accessible analytical kits and immunoassays have been developed to provide details on the format of the analysis. When it comes to food microbiology, immunochemical approaches are more laborious and time-efficient than microbiological methods, especially in strains where extraction and detection are laborious and time-consuming. By using various techniques, immunochemical processes enable a significant reduction of these needs.

Mycotoxins, the most dangerous and difficult-to-analyze group of food-related toxins, are routinely identified using immunoassays. Major mycotoxins have maximum permissible levels defined globally in several commodities due to their toxicity and their ubiquity in processed as well as raw food. As a result, immunoassays to detect controlled mycotoxins have been developed. Depending on the need, ELISAs and other tests are commercially available, with the list being updated every day.

ELISAs are frequently used in mycotoxin analysis to screen a lot of samples, while chromatographic techniques form the basis for confirmatory results. The development of immunoanalytical platforms enables the simultaneous identification of several chemicals and has been pushed by the introduction of MS and its multi-residual analytical features. Due to the great specificity and precision of immunoassays, it is frequently probable to completely omit time-consuming purification processes and reduce sample size as well as the volume of extracting solvents. However, immunochemical cross-reactivity may partially affect the assay's selectivity in the specific situation of tiny molecules with identical chemical properties, making it more ideal for screening than for a single compound's quantification. Immunoassays for pollutants found in industries, phytopharmaceuticals, and pesticides have recently gained acceptance as ways to supplement conventional analytical techniques in the food analysis domain. For pesticides, kits are available which are both quantitative and semi-quantitative. An antibody made against a chemical's protein conjugate may cross-react with other structurally related molecules in the same class, however, with varying degrees of success. This can be used to quickly estimate the total number of pollutants in a particular food item (24).

### 3.5. Solid phase microextraction

Solid phase microextraction (SPME) is a solid phase extraction sampling method that uses a fiber coated with an extracting phase, which may be a liquid (polymer) or a solid (sorbent), to extract various analytes (e.g., both volatile and non-volatile ones) from various media, which may be in the liquid or gas phase. As long as equilibrium is attained, or in the case of a brief period of pre-equilibrium, with the aid of convection or agitation, the amount of analyte extracted by the fiber is proportional to its concentration in the sample. Ultra-performance liquid chromatography incorporated with high-resolution MS and SPME incorporated with MS have been used for phytonutrient and aroma profiling followed by a nutrient overview by GC-MS. A holistic insight into breast milk composition is important for health benefits that derive from breastfeeding and the composition of infant formulas based on a novel analytical approach, holistic profiling of Human Breast Milk (HBM) lipidomes. SPME and LC-MS have been developed to improve microextraction (25). A new extraction method allows a wide range of lipids to be extracted directly from his HBM samples quickly and easily. A lipid extraction protocol provides high lipidome without using toxic solvents such as chloroform. Searching of lipid database by quadrupole time-of-flight MS detects lipids in HBM. A headspace solid-phase microextraction-gas chromatography-time-of-flight-mass spectrometry method was developed for the profiling of apple volatile metabolites. The selected SPME method was applied to the profiling of four different apple cultivars using GC-EI-TOF-MS. The developed headspace solid phase differential combined with gas chromatography-mass spectrometry (HS-SPME) sampling method is fully automated and is useful for obtaining volatile substance fingerprints in fruit (26). An improved method based on HS-SPME/GC-MS has been proposed for the semi-quantitative determination of volatile compounds. Polydimethylsiloxane or divinylbenzene fibers were used to extract unsettled particles from the headspace of bread dough samples that were dispersed in an aqueous sodium chloride solution (20%) and stored for 60 min at a temperature of 50°C in surrounding conditions. By calibrating with extracts tailored to the matrix, the method's excellent linearity for the selection of volatiles from various chemical groups has been confirmed.

### 3.6. Microscopic techniques

The majority of foods represent heterogeneous systems, and their several structural components determine their mechanical, biological, and functional properties along with their stability. Using different microscopic methods, a variety of meals and their structural properties can be estimated on a large scale. All these methods allow researchers to gain better clarity regarding the relationship between food components. With the influence of certain specific structural constituents on the different functionality, the impact of composition and processing on a substance's physical properties, the scattering of microbes and their food surface interactions, as well as changes in the accessibility and structural characteristics of different compounds are bioactive during digestion. Multiple microscopic techniques are used to describe matrices of food. Optic, electron, and probe microscopy are the most common forms. The most effective microscope technique will be based on the food type and the objective of the investigation. The ability to discriminate between two nearby objects, the degree of magnification, the sample preparation requirements, and the method's impact on the structure of the food should all be considered when selecting an appropriate methodology. The utilization of a combined imaging approach, such as light microscopy and electron microscopy, often makes data analysis and interpretation simpler and more efficient (27).

Early uses of light microscopy in the food industry focused mostly on ensuring food safety, such as identifying microbiological contamination or adulterants in food products. With the development of optical microscopy by Olga Flint, it is now possible to employ LM to determine the microstructures and macrostructures of meals using methods such as selective staining and optical contrast. LM continues to be important in the field of food research because of its availability, low cost, ability to distinguish between food color, and versatility in the testing area, which permits the analysis of food samples, especially certain wet specimens in ambient nature. The different advanced techniques like correlation compared to methods have expanded their applicability (e.g., scanning electron microscopy [SEM]). Its main disadvantages are its poor resolution and depth of focus, especially in the settings of a microscope. The different image processing methods used digitally such as focus or z-stacking are widely used in confocal microscopy and are utilized in wide-field digital microscopy to improve the focusing parameter, albeit this frequently results in picture acquisition periods. In food applications, LM techniques such as bright-field microscopy, polarized microscopy, and fluorescence microscopy are widely used. Apart from these, the main apparatus used is a conventional bright field microscope. Most modern microscopes are accompanied by a digital camera, which facilitates the analysis of the micrograph images (28). It is simple to attach polarizing and fluorescence accessories to them. To determine the presence, structural organization, and spatial distribution of particular food components in a product, fluorescent microscopy uses the autofluorescence method on compounds inside a specimen or the addition of selective fluorescent probes. If the fluorophores are chosen properly, their radiation may be widely recognized in the black backdrop when light with a specific wavelength is utilized to excite the intrinsic or additional fluorophores. Many dietary components are autofluorescent such as some aromatic amino acids, pigments, phenolic compounds, antioxidants, and tastes. It is well known that fluorophores are frequently found in food compounds; synthetic fluorescent dyes are frequently chosen for microscopy because of their higher quantum yields and brightness, environmental sensitivity (i.e., how the environment's physical and chemical characteristics impact emission), and selective interactions with particular food components. Rhodamine B and fluorescein isothiocyanate (FITC) are used to non-covalently label starch. To improve selectivity toward food components, fluorescent probes are linked to either lectins or antibodies. They constitute lectins that are fluorescently labeled like Concanavalin A and can effectively stain a-linked carbohydrates. For carbohydrate labeling, green fluorescent protein (GFP) can be used in conjunction with carbohydrate-binding modules that have high selectivity for carbohydrates such as cellulose. Nile Red is a widely used fluorophore that is soluble in lipids for assessing the lipid distribution in solid, semi-liquid, and liquid foods (29). Biomolecules such as proteins, carbohydrates, and phospholipids, tagged with fluorophores, are linked covalently. When selecting the different strains, it is crucial to take into account potential autofluorescence sources in samples because overlapping in the emission makes data analysis more difficult.

Currently, the function of important structural components like protein aggregates, fat crystals, or polysaccharide fibers inside the food can be understood using electron microscopy, which is a crucial tool for assessing the external or internal composition of meals. Since electron microscopy produces images by using an accelerated electron beam rather than photons, it has a better resolution than LM. Due to their shorter wavelengths than visible light, electron beams can identify tiny structures. To reduce electron beam scatter, conventional electron microscopy operates in a high vacuum. Therefore, thorough sample preparation is necessary to remove water or other volatile compounds from the food samples. Artifacts are frequently introduced as a result, which affects the final photographs. The most used EM method for studying foods is SEM. A low-energy electron beam is used to scan the specimen's surface in an x-y direction, and the sample's electrons are detected using SEM. SEM gives a distinct three-dimensional perspective of the specimens enabling structural alterations to compositional or processing-related variances in bulk attributes. To test wet samples with cryogenic SEM, the specimens must be frozen before being tested while in a cryo condition connected with SEM. Thus, the images obtained are more accurate depictions of food systems than images made with conventional methods of SEM. For samples with high moisture content, like dairy products, this approach has been widely utilized. Transmission electron microscopy (TEM) images are produced by the abrasion of high and intense electron beams as it passes thin specimens that have undergone careful preparation. It can be difficult to interpret the data from the 2D TEM micrographs; instead, they need to be viewed in the context of the 3D environment being investigated. In the TEM, components with different electron densities can be estimated such as the myofibrils of pig muscle rupture as a result of ultrasonic cavitation during the curing process. The development of environmental scanning electron microscopy (ESEM) and environmental transmission electron microscopy (ETEM), which permits the testing of hydrated samples in their natural state in a gaseous environment with less sample preparation, has significantly increased the use of electron microscopy in food applications in recent decades. Compared to conventional SEM and TEM methods, environmental EM generates images utilizing minimal artifacts but lower resolution. E-SEM is used successfully for tracking the formation of colloidal aggregates and films in food models. Conventional optical microscopy that is fitted with lasers and Raman detectors can be used to perform Raman microspectroscopy. For example, the mapping of the chemical composition of a food sample and material distribution as to how they are altered by processing is made possible by combining microscopy and Raman detection. The spectrum is captured at each position along the specimen's spatial range as the surface is scanned point-by-point or line-by-line. The banding pattern within a region of the spectrum allows the identification of proteins and carbohydrates, thereby utilizing sophisticated chemometric methods. For each inspected spot, the intensity of a band is plotted and related to its concentration to generate images. With the advent of affordable and simple equipment with specialized software for processing spectra and images, Raman microspectroscopy becomes more accessible. The minute examinations of food samples have substantially advanced in recent years. The application of molecular modeling approaches will continue to support this ongoing trend. Chemical imaging methods will advance since they are non-invasive and relatively easy to use. They will be able to link microstructural alterations to compositional changes as a result, which will aid in the logical design of innovative meals and the optimization of processing techniques.

### 3.7. Nutritional metabolomics

**Figure 5:**
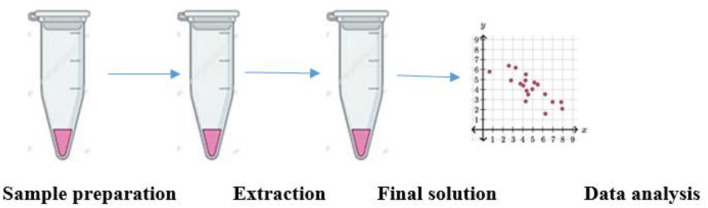
Nutritional metabolomics. A typical flowchart describing the application of metabolomics in the field of nutrition. The most important step of sample preparation is followed by the extraction of the cell and leading to the formation of the final solution. An *in silico* data analysis generates results, thereby providing valuable output.

Metabolomics studies consistently show that alterations in fatty acid, lipid, and tryptophan pathways are common and associated with disease state and outcome in critically ill patients. Metabolomics offers many opportunities to advance nutritional cancer epidemiology. This statement overview summarizes recent research, challenges, and prospects in nutritional metabolomics and epidemiological cancer research (Figure 5). Additional metabolomics research examining the relationship between food exposures and cancer risk, prognosis, and survival is required, in addition to methodological research, longitudinal analysis, and studies validating biomarkers. Although the objective remains, metabolomics offers a potential route for future research in nutritional cancer. In addition, nutrients that directly and indirectly affect and regulate gene activity play important roles in the prevention and treatment of chronic degenerative diseases. A growing body of research has found that obesity and altered metabolic responses to low-calorie weight-loss diets can be altered by genetic variants associated with obesity, metabolic status, and nutritional preferences. Survival depends on the intake of essential nutrients, and dietary components influence both disease prevention and promotion. Metabolomics is the study of all low molecular weight metabolites in a system. Nutrients and dietary components are key environmental factors that interact with genomes, transcriptomes, proteomes, metabolomes, and microbiota, and this lifelong interaction defines an individual's health and disease. In genetically predisposed people who are exposed to environmental factors, rheumatoid arthritis is a chronic autoimmune disease that causes a systemic immune-inflammatory response. In recent years, increasing evidence suggests that dietary factors and gut microbiota play a central role in the risk and progression of rheumatoid arthritis. Plasma samples obtained from lactating women participating in a study in Samar, Philippines, with low (vitamin A–) or adequate (vitamin A+) (plasma retinol < 0>) vitamin A status were 1.05 μmol/was chosen). A total of 28 metabolites were altered in vitamin A– and vitamin A+ status groups, and 24 were lipid mediators (*P* = 0.05). Low quantities of oxylipins produced from arachidonic acid, eicosapentaenoic acid, and the VA group's lysophospholipids and sphingolipids were among these lipid mediators (*P* < 0.05). Reduced lipid mediator concentrations were found in multi-assay dietary profiles of low and adequate vitamin A status in breastfeeding women. Diet has an impact on how the gut microbiota and its mammalian host interact. Small chemicals produced by microbiota may be ingested by the host and affect a variety of crucial physiological functions (30).

## 4. Protocol for nutritional profiling

A very-low-calorie ketogenic diet contains low carbohydrates. The cardiovascular risk profile is distinguished by the presence of abdominal obesity, high total cholesterol, high triglycerides, and abnormal fasting blood glucose levels. Irritable bowel syndrome (IBS) is a chronic functional gastrointestinal disorder characterized by abdominal pain associated with bowel movements or changes in bowel habits. How different diets modulate gut microbiota profile such as a low-FODMAP diet, which is effective in people with IBS, contributes to changes in the gut microbiota. The purpose of this review was to examine different dietary protocols (conventional dietary advice, low FODMAP diets, gluten-free diets, etc.). Although there is no ideal nutritional protocol for patients with IBS-D, we investigated the impact of different nutritional approaches on gut microbiota composition to better define efficient strategies to treat these disorders. It seems important to consider. Alterations in the gut microbiome have been shown to contribute to the progression of metabolic diseases such as prediabetes and type 2 diabetes. Studies suggest that *in vivo* modulation of the gut microbiota by certain probiotic microbes can improve insulin sensitivity and glycemic control, and prevent or delay the onset of type 2 diabetes. However, further research is needed to understand the efficacy of probiotics as a treatment for metabolic diseases. Evidence-based multi-probiotics are designed to shift a cohort of gastrointestinal bacteria from prone to balanced in order to improve metabolic markers associated with type 2 diabetes. A total of 60 adults with prediabetes or type 2 diabetes (diagnosed within the last 12 months) and BMI ≥25 kg/m^2^ will be enrolled in a double-blind, placebo-controlled pilot study. Participants will be randomized to multiple probiotics or placebos for 12 weeks. Both groups receive lifestyle and nutrition advice. The primary endpoint was the change in fasting plasma glucose between groups from baseline to his 12th week. Secondary outcome parameters include changes in lipid profile, systemic inflammation, intestinal permeability, and stool microbial and metabolomic profiles. Blood and stool samples will be collected at baseline and 12 weeks after treatment. Research on the role of vitamin C in the prevention and treatment of pneumonia and sepsis has been ongoing for decades (31). This study provided a strong basis for extrapolating these findings to patients with severe COVID-19. Studies have shown that patients with pneumonia and sepsis have low vitamin C status and increased oxidative stress. Giving vitamin C to patients with pneumonia can reduce the severity and duration of the disease. Severely ill patients with sepsis should be given intravenous gram doses of the vitamin to normalize plasma levels. This is an intervention that several studies have found to reduce mortality. Vitamins have multiple physiological functions, many of which are relevant to COVID-19. These include antioxidant, anti-inflammatory, antithrombotic, and immunomodulatory functions. Preliminary observational studies have shown low vitamin C status in critically ill patients with COVID-19. Several randomized controlled trials (RCTs) are currently registered worldwide, investigating intravenous vitamin C monotherapy in patients with COVID-19. Studies were conducted in populations with chronic vitamin C deficiency because vitamin C deficiency is common in low- and middle-income settings, and many of the risk factors for vitamin C deficiency overlap with those for COVID-19, which may have a greater effect. This is particularly relevant to global research efforts, as COVID-19 disproportionately affects lower-middle-income countries and low-income populations around the world. A small study from China was completed prematurely, and the results are now being reviewed by experts. Patients who received vitamin C therapies tended to be more seriously unwell. Mortality was significantly reduced. Future results from a large ongoing RCT will provide more conclusive evidence for the optimization of intervention protocols in future studies. Early and sustained dosing is warranted as it may improve efficacy. Patients with vitamin C deficiency are affected by respiratory infections due to its superior safety profile, low cost, and potential for rapid scale-up of production (Table 2).

**Table 2:** Techniques currently used in nutritional profiling of foods with advantages and disadvantages.

**Technique**	**Use in nutritional profiling**	**Advantages**	**Disadvantages**
Gas Chromatography (GC)	Detects compounds like sterols, oils, low-chain fatty acids and contaminants like pesticides and pollutants	Allows separation of the components of complex mixtures in a reasonable time owing to high level of efficiency	Limited to thermally stable and volatile compounds
High-Performance Liquid Chromatography (HPLC)	Identifies major and minor sugars in carbohydrate research as well as vitamins	Rapid and precise method for identification of specific chemical compounds	Expensive and complex process which is not applicable to work for all samples
Nuclear Magnetic Resonance (NMR) Spectroscopy	Determines water content, metabolic composition including presence of flavonoids, organic acids and soluble sugars	Aids in obtaining clear structural information of molecules in their natural environment	It is not a cost effective method and determination of higher molecular weight structures poses a problem
Atomic Absorption Spectroscopy (AAS)	Quantifies trace elements such as iron, sodium, calcium	Easy operation and high level of sensitivity as well as accuracy	Non-metals cannot be identified by this method
Mass Spectrometry (MS)	Measures protein concentration, trace elements and unknown metal species	Automated technique that can be employed on a large scale	Identification of hydrocarbons having similar ions is difficult
PCR	Detects dietary allergens like peanuts, buckwheat and microbes and unwanted contaminants	Highly sensitive technique producing quick results	Cannot be utilized for amplification of unknown targets
ELISA	Analysis of mycotoxins and other pollutants present in food	High precision and rapid results	Quite a labor-intensive as well as expensive method to carry out
Solid Phase Microextraction (SPME)	Used for phytonutrient and aroma profiling as well as determination of volatile compounds	It is a simple and economic method to implement	Limited choice of selectivity
Microscopic Techniques	Estimation of structural properties of food components like protein and fats	High resolution helps in better viewing	Quite expensive instruments
Nutritional Metabolomics	Identifies nutrients, dietary fibers and metabolites and their interaction with genomes defining human health and diseases	Useful tool for premature disease diagnosis providing quick analysis	Low in sensitivity

## 5. Case studies of nutritional profiling using chromatography

### 5.1. Case study on edible vegetable oils

One of the main categories of food items are oils and fats, with edible vegetable oils generally replacing animal fats in processed food compositions. Their validity has, therefore, become a crucial concern (32). The identification of their adulteration can be facilitated by fat and oil authentication using the examination of several component lipid classes by both HPLC and GC analyses. Triacylglycerols, fatty acids, sterols, and other minor substances are found in edible vegetable oils (33). The whole fatty acid family of edible fat, or “fatty acidomics,” is a crucial indicator and quality measure for these food matrices. After being converted into fatty acid methyl esters (FAMEs), they are often subjected to GC-MS analysis (33). Their identification is performed by comparing their mass spectra to the retention indices of standards and/or by utilizing mass spectra libraries. To determine if there has been adulteration, the percentage values for each fatty acid are compared to those accepted by national and international organizations. An increase or reduction in the quantities of certain fatty acids may be indicative of this. Even if they are not very unique, fatty acid patterns may be beneficial for determining authenticity, particularly when examining pure oils. For instance, the presence of palm oil is indicated by the presence of significant percentages of palmitic acid, whereas the presence of rapeseed oil is indicated by the presence of trace levels of erucic acid. The FA composition is too complicated to be utilized for verification in blends including oils from several botanical sources, in part because of chromatographic peak merging. It is acknowledged that the fatty acid profile only makes up a small portion of the composition of oils; occasionally, further examination of other macrocomponents and microcomponents of oils is required (34).

### 5.2. Case study on milk and dairy products

By analyzing the lipids in dairy products, both qualitatively and quantitatively, it is possible to identify foreign fat in milk fat. Butter is the most significant milk fat product due to its extensive usage. Its quality is crucial because of this; it has always been tainted by the addition of less expensive vegetable or animal fats. By examining fatty acids (FAs), triacylglycerols, and minor lipid components of the unsaponifiable fraction, foreign fat in milk fat can be found (35). Following GC analysis, the addition of vegetable oils to milk fat can be identified by measuring individual FAs or the concentration ratio of two or more FAs. FA ratios have also been applied in the past to distinguish between various types of milk fat (36). Classes of substances with equal numbers of acyl-C atoms have been suggested for and put to use in TAG analysis by utilizing GC analysis (35). For authentication reasons, analysis of small chemicals can be quite helpful. By using silica column GC to analyze free sterols or trimethylsilyl derivatives, it was possible to identify the presence of vegetable oils in milk fat that had been tampered with (37). For the qualitative and quantitative study of the unsaponifiable fraction of milk lipids (cow butter, buffalo, sheep, and goat milk), a thorough GC technique with dual MS/FID detection was developed and refined. The identity of chemicals that were found was confirmed using a GC-high-resolution TOF-MS analysis. The efficacy of such a technology for dairy product authenticity tests was illustrated by the high number of chemicals that were isolated and identified (38).

### 5.3. Case study on honey

The adulteration of honey is a complicated issue that currently has negative effects on nutrition and organoleptic quality as well as considerable economic impact. Numerous forms of economically driven adulteration have been discovered in the honey business; chromatographic techniques, less-priced syrups, and misleading claims about the botanical and geographic origins of honey have all been implicated. Numerous writers have suggested using chromatographic methods to analyze the primary components of honey, which are carbohydrates. Corn syrup (CS), high-fructose corn syrup (HFCS), invert syrup (IS), and high-fructose inulin syrup are examples of common, affordable sweeteners that are added to honey as adulterants (HFIS). A high-performance anion-exchange chromatography pulsed amperometric detection (HPAEC-PAD) system (39), HPLC equipped with a common RID detector, and simultaneously GC-FID and HPAEC-PAD analyses were used to obtain a fingerprint profile of honey oligosaccharides (40, 41). This analysis used a straightforward HPAEC-PAD method for quantitative analysis of maltooligosaccharides to identify honey adulterated with CS and HFCS. The technique was based on fractionating honey carbohydrates using activated charcoal sample treatment before analyzing the results. The amount of oligosaccharides was measured after analysis of several samples of honey, CS, and HFCS. A honey sample was made into adulterated samples by adding 5, 10, and 20% of syrup to it. The technique proved to be a helpful tool for identifying honey tainted with commercial syrups. Fructose and glucose were measured using the HPAEC-PAD technique, and the full profile of disaccharides and trisaccharides was determined using the GC-FID method (41). With the use of statistical processing, the outcomes of the two procedures were integrated. This strategy was developed for a better understanding of the effect of syrup when added to acacia, chestnut, and lavender honeys. Using genuine honey samples, an oligosaccharide database was produced. Numerous French monofloral commercial honeys were examined. For the examined monofloral honeys, the limits of detection were quite good, ranging between 5 and 10%.

### 5.4. Case studies with fruit juices

Due to their taxonomic uniqueness, phenolic chemicals are particularly promising indicators for determining the authenticity of the food. Orange juice adulteration has been a particular focus of their chromatographic investigation. Juices from sweet oranges (*Citrus sinensis*), whose consumption has greatly grown recently, might also contain grapefruit (*Citrus paradisi*), tangerine (*Citrus reticulata*), or lemon (*Citrus limon*). HPLC-DAD/ESI-MS/MS and HPLC-DAD were developed by Abad-Garcia et al. (40) for the characterization and quantification of phenolic chemicals in citrus juices, respectively (42). These phenolic chemical profiles were examined with the goal of distinguishing citrus liquids according to the species utilized for their elaboration: sweet orange, tangerine, lemon, or grapefruit. They are typical of the Spanish citrus fruit juice production. For the purpose of creating classification models and identifying potential markers, statistical and chemometric techniques were used. All delicious orange and tangerine liquids were successfully recognized by classification models. Despite needing an outside validation, the proposed model appears to be effective at identifying sweet orange juice tainted with tangerine juice.

## 6. Case study of nutritional profiling with NMR

Despite being a relatively new use of NMR, food authentication accounts for the bulk of NMR's applications in the field of food science (43). Beverages, fruits and vegetables, honey, fats and oils, spices, dietary supplements, as well as meat, fish, and dairy products, are some examples of the applications. As these are the most frequent causes of fraudulent claims for food authenticity, the elements that are analyzed most frequently include variety, geographic origin, harvest season and/or agronomic techniques, and adulteration with items of lower price and quality (44). The typical nucleus used for analysis is the proton. Exceptions include the application of ^13^C NMR-based metabolomics for coffee authentication to avoid interactions between caffeine and chlorogenic acids that cause issues related to chemical shift alignment and the application of ^13^C NMR coupled with discriminant analysis for the classification of olive oils (45). Numerous uses of ^31^P NMR spectroscopy in food authenticity exist, primarily in the field of olive oil analysis (46). Food adulteration by melamine, adulteration of honey by syrups (high fructose corn, maltose, or jaggery syrup), adulteration of olive oil by vegetable oils or lampante/pomace olive oils, mixing of ground black pepper with buckwheat and millet, adulteration of culinary spices by Sudan I dye, and meat adulteration are typical examples of spectroscopic methodologies used for food authentication. There have been reports of the authenticity of milk, olive oils, honeys, wines, spirits, spices, and other culinary items, as well as saffron and lentil seeds (47, 48).

Moreover, using SNIF-NMR, which is site-specific, it is possible to accurately fingerprint natural compounds. The identification of the geographic origin of the wine, pioneered by the EU in 1990, is a well-known use of SNIF-NMR. Food's geographic origin has been evaluated using profiling techniques including non-targeted ^1^H-NMR analysis. NMR analysis has been used to determine adulteration, including the addition of cane or corn sugar to maple syrup, the adulteration of red wine with anthocyanins, and artificial tastes marketed as natural. Wines, coffees, olive oils, honeys, fish, spirits, vinegars, and saffron are among the foods that may be differentiated by their metabolic profiles using NMR (49).

## 7. Metrology in nutritional profiling of food

It is often necessary to include metrological principles with chemical and biological measures in food analysis. Based on the definition of metrology as “the science of measurement, embracing both experimental and theoretical determinations at any level of uncertainty in any field of science and technology,” its concepts include the definition and implementation of globally recognized units of measurement and (metrological) traceability by evaluating uncertainty in relation to national and global reference standard measurements. In addition, metrology offers the resources needed to make measurement findings consistent and comparable.

Only a small number of techniques can use the RMs that are now available since they are only approved for a small number of parameters and small numbers of matrices. To meet the new requirements related to the analytical determination of nutraceutical substances and natural substances with protective effects on health, new RMs are either required or closely related to the new analytical needs and the emerging challenges of food safety (e.g., application of nanotechnologies or biotechnologies). The adoption of new RMs to be used for ensuring the origin and traceability of raw materials and products as well as for identifying frauds and adulterations is associated with a particularly urgent necessity (50). According to this perspective, new RMs are becoming more important in order to recognize genetic markers and chemical compositions and to confirm the geographical and/or biological (botanical, zoological, and genetic) origin of raw materials and finished goods. The ability to create multiparameter RMs for use in multi-parametric determinations, qualitative analyses, and identity studies through the definition of elemental, isotopic, molecular, and/or genetic markers or patterns for the traceability of food products is particularly intriguing, especially with regard to assessing the foodome (51). In addition to promoting multiparametric characterization standardization, a more thorough characterization of the foodome present in these materials would be of additional value for laboratories to avoid the costs of acquiring different materials for the required set of analytes and for RM producers to reduce the effort, for example, for the testing of homogeneity.

In addition to the use of RMs, accurate calibration of equipment, particularly the important components, is necessary to ensure the quality and traceability of measurement data. As a result, it is important to develop and maintain the traceability chain, which is described as a series of standards and calibrations that connect a measurement's result to a reference.

There is a significant dispersion in Europe and around the globe when it comes to the condition of food analysis and research today. Despite the fact that food production, marketing, and fraud occur on a multi-national and even international scale, every European member state approaches the overall goal of verifying food integrity differently. This is in addition to the common European Commission regulations on official food controls and common notifications within the EU Rapid Alert System for Food and Feed (RASFF) (52). Furthermore, a wide range of scientific fields and organizations are involved. It is worse that the information produced by these control and verification procedures is dispersed and entered into a wide range of diverse databases. Many initiatives seem to be unnecessary since they are being repeated and gaps are not being consistently filled.

## 8. Conclusion

This review mainly provides an outline that focuses on the nutritional model and the several bioanalytical tools that are used for detection and their potential applications. Recent scientific literature and evidence show that the classification of foods depends not only on the nutrition composition but also on the distribution of food in our total diet. To take into account the significance of the food in the diets of the general population, children, and other particular groups, nutrient profile models are also required. NP models are developed to prevent diseases that are still prevalent such as obesity, vitamin and mineral deficiencies, etc. Based on research, GC/MS and HPLC are used for the characterization of the food of interest and can also be used to evaluate the quality, aroma, and authentication of food samples. In addition, the GC method also has some drawbacks which can be further improved by optimizing parameters. Microscopic analysis showed significant advancements in detecting food quality and nutrition values and providing food security globally and high-quality standards of foods. PCR and different immunoassay techniques facilitated the analysis of food offering possibilities for the detection of allergens and toxins, thereby increasing food hygiene. On the other hand, nutritional metabolomics was proposed across populations addressing nutritional phenotypes and health and diseases but the rapid development of improved biosystem models and bioanalytical techniques helped to advance nutrition research. There are currently numerous initiatives underway to create, verify, and test nutrient profile models. The Food Standards Agency (FSA), the UK's counterpart of the Food and Drug Administration (FDA) in the US, has released interim and final findings, and they are now accessible online. France and the Netherlands have both developed additional NP models. These models often include both nutrients and dietary groups and are based on certain combinations of macronutrients, vitamins, and minerals. The relationship between nutrition and health is validated by the existence of nutritional databases. In the future, governments, industries, and other non-profit organizations should develop such advanced models with additional studies and research for more appropriateness making the domain of global health a priority.

## Author contributions

DM, UC, MB, and SG contributed equally in writing the manuscript. DK revised, corrected, and edited the manuscript. All authors contributed to the article and approved the submitted version.
